# The risk of systemic lupus erythematosus associated with Epstein–Barr virus infection: a systematic review and meta-analysis

**DOI:** 10.1007/s10238-018-0535-0

**Published:** 2018-10-25

**Authors:** Zhao-Xia Li, Shan Zeng, Hui-Xia Wu, Yi Zhou

**Affiliations:** Department of Rheumatology, The First Affiliated Hospital, Jinan University, No. 613 West Huangpu Ave, Tianhe District, Guangzhou, 510630 China

**Keywords:** Epstein–Barr virus, Systemic lupus erythematosus, Meta-analysis

## Abstract

Previous systematic reviews have found a higher sero-prevalence of EBV antibodies in SLE patients compared with controls. Because many studies have been published, there is a need to apply more precise systematic review methods. We examined the association between EBV and SLE patients by conducting a systematic review and meta-analysis of case–control studies that examined the prevalence of EBV antibodies and the DNA-positive rate. We searched the MEDLINE and EMBASE databases from 1966 to 2018 with no language restrictions. The Mantel–Haenszel odds ratios (OR) for EBV antibody sero-positivity were calculated, and meta-analyses were conducted. Quality assessment was performed using a modified version of the Newcastle–Ottawa scale, and 33 studies were included. Most studies found a higher sero-prevalence of VCA IgG and EA IgG in SLE patients compared with controls. Meta-analysis demonstrated a significantly higher OR for sero-positivity to VCA IgG and EA IgG for SLE cases (2.06 [95% confidence interval (CI) 1.30–3.26, *p* = 0.002] and 7.70, [95% CI 4.64–12.76, *p* < 0.001], respectively). The overall OR for the DNA-positive rate for SLE patients compared with controls was 3.86 (95% CI 1.52–9.83, *p *= 0.005). Other antibodies, i.e., VCA IgA/IgM, EBNA IgA, and EA IgA/IgM, also demonstrated a significant difference between SLE patients and controls. These findings support previous systematic reviews; however, publication bias cannot be excluded. The methodological conduct of studies could be improved, particularly when selecting controls and analyses of laboratory conduct.

**Electronic supplementary material:**

The online version of this article (10.1007/s10238-018-0535-0) contains supplementary material, which is available to authorized users.

## Background

Systemic lupus erythematosus is an autoimmune disease without clear pathogenesis. This disease is characterized by polyclonal B cell activation and altered T cell function with the presence of multiple autoantibodies and impaired cell-mediated immunity. It is believed that both genetic and environmental factors contribute to disease development [1]. Bacteria and virus infections are major important environmental factors that may be initiated and involved in the pathogenesis of SLE. The Epstein–Barr virus (EBV) is of particular interest. It has been reported that Epstein–Barr nuclear antigen-1 (EBNA-1) has a high degree of homology with some proteins that cause an autoimmune humoral response [2, 3]. This molecular mimicry may play an essential role in the induction of antibodies by EBV infection in SLE patients. Recently, Harley et al. [4] reported that nearly half of SLE risk loci are occupied by the EBNA-2 protein and many co-cluster with transcription factors, providing an essential new perspective on the mechanism of SLE pathogenesis.

The first positive association between EBV infection and SLE was found in 1971 [5]. Since then, many researchers have used various angles to investigate the possibility of this link. However, previous studies have failed to detect a consistent association. The first and only systematic review that updated on the association between SLE and sero-positivity for different EBV antibodies was that of Hanlon et al. [6]. These authors found a statistically significant higher sero-prevalence of viral capsid antigen (VCA) IgG but not EBNA1 in cases compared with controls. Meta-analyses for early antigen (EA)/D IgG and VCA IgA also significantly demonstrated higher ORs in cases compared with controls.

Many studies have since been published and may help in more fully estimating the association. In addition, some authors postulated that the increase in antibodies in SLE was brought about by generalized immune hyper-reactivity in lupus rather than by any specific property of the EBV. It was later thought that the best way to clarify this question would be at the DNA level [7–10]. Therefore, we undertook this meta-analysis with a broader, more comprehensive search strategy with no language restriction including both the sero-prevalence of antibodies and the EBV DNA load.

## Methods

We conducted a systematic literature search of MEDLINE (source: PubMed, January 1, 1966 to June 20, 2018) and EMBASE (January 1, 1974 to June 20, 2018) using text and keywords in combination as MeSH terms and text words (Additional file: Table S1). We searched articles published without language restrictions and scrutinized the references from these studies to identify other relevant studies.

### Study selection

To minimize differences between studies, we imposed the following methodological restrictions for the inclusion criteria: (1) The study had to be a cohort or case–control study of recruited patients with an SLE diagnosis and controls with no SLE diagnosis (healthy or unhealthy). (2) Patients could be from any age group, and studies that assayed the EBV DNA genome using peripheral blood mononuclear cells (PBMCs) and/or serum or antibodies (using any method) for any of the following EBV antigens: VCA, EA, EBNA-1, or 2. Exclusion criteria were as follows: (1) non-human studies. (2) Studies only measuring IgM antibodies. (3) Studies that reported serum antibodies without a specified antigen or an antigen without a specified antibody type.

### Quality assessment

Because all of the included studies were non-randomized, we used the Newcastle–Ottawa scale (NOS) to access the quality of the studies [11], which was adapted to award two stars for blinded blood sample analysis, one star for conducting the analysis in a clinical laboratory (independent from the investigators), one star for specifying explicit laboratory cutoffs for sero-positivity, and one star for reporting the presence or absence of missing data.

### Data analysis and statistical analysis

Two investigators (Li ZX and Zeng S) independently extracted data from eligible studies and reached a consensus for all items. For all studies, we extracted the following: the name of the first author, year of publication, country where the study was performed, number of patients, study population, gender category, selection criteria for control participants, methods for outcome assessment, and reported adjustments for potential confounders. The percent agreement between two authors on the review quality ranged from 85 to 100%.

The meta-analysis was performed by STATA 12.0 (Stata Corp, College Station, TX). We pooled estimated Mantel-Haenszel ORs from each study separately for each outcome using a random-effects meta-analysis. We evaluated the statistical heterogeneity of the ORs by calculating the Cochrane Q statistic (significance level: *P* ≤ 0.1) and the *I*^2^ statistic, applying the following interpretation for *I*^2^: < 50%, low heterogeneity, 50–75%, moderate heterogeneity, and > 75%, high heterogeneity [12]. The probability of publication bias was assessed by visual inspection of a funnel plot and Begg’s test [13, 14]. If the funnel plot and Begg’s test revealed asymmetry, we performed the Duval and Tweedie nonparametric “trim and fill” procedure to assess potential effects of publication bias [12]. This method considers the possibility of hypothetical “missing” studies that might exist, imputes their ORs, and recalculates a pooled OR that incorporates the hypothetical missing studies as though they exist. Subgroup analyses and meta-regression models were performed to investigate potential sources of between-study heterogeneity.

## Results

A total of 2000 references were retrieved using our initial search algorithm, and of these, 33 studies were ultimately included (Additional file: Figure S1). One study was in Turkish [15], one in Chinese [16], one in French [17], and the others were in English. All of the reports were case–control studies published between 1988 and 2018. There were two pediatric studies and 31 adult studies. The characteristics of the 33 eligible studies are listed in Table 1.

**Table 1:** Characteristics of included studies

Study ID	Matching	Location	Case					Controls					Anti-EBV(IgG/IgA)/ DNA load	Test used
			Total	Source/type	Female	Male	Age^a^	Total	Source/type	Female	Male	Age^a^		
Berkun 2009 [18]	Age and geographically matched	Colombia	120	1982 ACR criteria for SLE	119	1	38.6 (11.9)	140	Healthy controls	130	10	39.1 (10.1)	VCA, EBNA1, EA (IgG)	IFA
Chen 2005 [19]	Sex and age-matched	Taiwan	36	1997 ACR criteria for SLE	32	4	30.7 (6.5)	36	Healthy controls	32	4	30.6 (6.2)	VCA (IgG/IgA/IgM)	IFA
Chen 2010 [20]	Sex and geographic location	Taiwan	94	1997 ACR criteria for SLE	82	12	42.1 (9.8)	370	“Healthy volunteers.”	220	150	35.7 (13.9)	VCA (IgG), EBNA1 (IgG/IgA), DNA	ELISA
Esen 2012 [21]	None	Turkey	198	1997 ACR criteria for SLE	180	18	38 (13)	65	Not specified	42	23	35 (7)	VCA (IgG), EBNA1 (IgG), EA/D (IgG)	IFA
Huggins 2005 [22]	None	UK	36	1997 ACR criteria for SLE	36	0	45 (14)	25	Blood donors	25	0	47 (18)	VCA, EBNA1, EA (IgG)	IFA
James 1997 [8]	“Similar by age ± 10 years.”	USA	117	1982 ACR criteria for SLE	NA	NA	15.8 (2.15)	153	Siblings/community controls	NA	NA	15.4 (2.51)	VCA (IgG)	ELISA
James 2001 [23]	“Similar by age.”	USA	196	1997 ACR criteria for SLE	184	12	44.7 (12.4)	392	Selected from pedigrees from lupus genetic study	368	24	45.9 (12.9)	VCA (IgG)	ELISA
Kitagawa 1988 [24]	None	Japan	65	1982 ACR criteria for SLE	NA	NA	NA	66	“Healthy donors.”	NA	NA	NA	EBNA1 (IgG)	IFA
Lau 1998 [9]	Age and sex-matched	Hong Kong	34	1982 ACR criteria for SLE	NA	NA	NA	22	Not specified	NA	NA	NA	VCA, EA (IgG/IgA)	IFA
Lu 2007 [25]	Age, sex, living place	Taiwan	93	1997 ACR criteria for SLE	88	5	35.2 (14.2)	370	“Healthy volunteers.”	351	19	NA	EBNA1 (IgA), anti-EBV-DNASE (IgG), DNA	ELISA, PCR
Marchini 1994 [26]	None	Italy	40	Patients attending the Clinical Immunology Unit	NA	NA	NA	20	NA	NA	NA	NA	EBNA1 (IgG)	ELISA
Newkirk 1996 [27]	None	Canada	70	1982 ACR criteria for SLE	63	7	44.3 (2.5)	31	Normal individuals	19	12	46.5 (2.8)	EA (IgG)	ELISA
Ngou 1996 [28]	None	France	33	1982 ACR criteria for SLE	NA	NA	NA	50	Blood donors	NA	NA	NA	EBNA1 (IgG)	IFA
Parks 2005 [29]	Age-matched by 5-year	USA	230	1997 ACR criteria for SLE	207	23	NA	276	Community controls	248	28	NA	VCA (IgG/IgA/IgM)	ELISA
Stratta 1999 [30]	None	Italy	60	1982 ACR criteria for SLE	51	9	41/21 to 60	100	Blood donors	28	72	39 (15)	VCA, EA (IgG)	IFA
Tazi 2009 [17]	Age-matched	Morocco	44	1997 ACR criteria for SLE	39	5	33/19 to 55	44	Blood donors	39	5	33/19 to 55	VCA, EBNA1 (IgG)	ELISA
Tsai 1995 [10]	Age-matched	Taiwan	16	1982 ACR criteria for SLE	NA	NA	16.9 (3.3)	20	NA	NA	NA	12.3 (2.6)	VCA (IgG)	IFA
Us 2011 [15]	None	Turkey	50	1997 ACR criteria for SLE	NA	NA	33 (12)	50	Blood donors	NA	NA	35 (14)	VCA, EBNA1, EA (IgG)	ELISA
Westgeest 1989 [31]	None	Netherlands	14	1982 ACR criteria for SLE	14	0	NA	84	Blood donors	NA	NA	NA	EBNA1 (IgG)	IFA
Yokochi 1989 [32]	None	Japan	16	1982 ACR criteria for SLE	16	0	53 (12)/27 to 72	30	Healthy controls	26	4	46 (9)	VCA, EBNA1, EA (IgG)	IFA
Zhang 1999 [16]	None	China	36	“SLE”	NA	NA	NA	45	Normal controls	NA	NA	NA	VCA (IgG/IgA)	IFA
Draborg 2012 [33]	None	Denmark	60	1997 ACR criteria for SLE	55	5	39.4/21 to 76	20	Healthy volunteers	12	8	32.1/25 to 63	VCA (IgG/IgA), EBNA1 (IgG), EA/D (IgA, IgG, IgM)	ELISA/ IFA
Chougule 2017 [34]	Age and sex	India	87	1997 ACR criteria for SLE	78	8	28(10)	50	Healthy controls	45	5	29(5)	VCA (IgM, IgG), EBNA (IgG)	ELISA
Csuka 2012 [35]	None	Hungary	301	SLE	275	26	44.0/37.0 to 57.0	345	VOLUNTEERS and 238 parents of patients scheduled for bone marrow transplantation	189	156	46.0/37.0 to 57.0	EBNA-1 (IgG)	ELISA
Vista 2017 [36]	Sex and age ± 5 years	Filipinos	233	1982 or 1997 ACR criteria for SLE	219	14	29(12)	764	Unaffected first-degree relative/unrelated controls	337/152	206/69	41(17)/27 (7)	VCA, EA, EBNA-1 (IgG)	ELISA
Broccolo 2013 [37]	Matched for age, gender and living area	Italy	22	SLE	22	0	54 (18)	58	“Healthy volunteers.”	47	11	55 (15)	VCA, EA, ENNA, DNA	ELISA PCR
Han 2018 [38]	Similar demographic	China	116	1997 ACR criteria for SLE	104	12	31.7/15 to 67	76	“Healthy volunteers.”	68	8	29.8/18 to 47	VCA, ENNA	ELISA,
YU 2005 [39]	Age- and sex-matched	Taiwan	87	1997 ACR criteria for SLE	80	7	35.3 (9.7)	174	Healthy controls	160	14	34.8 (10)	DNA	PCR
Rasmussen 2015 [40]	None	USA	77	1997 ACR criteria for SLE	71	6	38/20 to 76	29	Healthy controls	22	7	42/23 to 63	EA/D (IgG/IgA/IgM)	ELISA
Draborg 2014 [41]	Sex- and age-matched	Denmark	22	1997 ACR criteria for SLE	21	1	42.6/20 to 81	22	Volunteers	20	2	39.1/25 to 59	EBNA1, EA/D (IgG, IgA, IgM)	ELISA
Draborg 2016 [42]	Sex- and age-matched	Denmark	27	1997 ACR criteria for SLE	26	1	42.4/21 to 81	27	Healthy controls	25	2	37.2/22 to 61	EBNA1 (IgG)	ELISA
Martin 2011 [43]	Age- and sex-matched	France	118	1997 ACR criteria for SLE	107	11	34.5/16 to 61	31	Healthy controls	27	4	33.0/19 to 57	anti-EBV IgG (unspecified), DNA	ELISA, PCR
Uk 2004 [44]	None	Korea	66	1997 ACR criteria for SLE	NA	NA	45.7(15.6)	63	Healthy volunteers	NA	NA	38.5 (10.8)	DNA	PCR

*NA* not specified

^a^Mean (standard deviation)/or range

The features of the participants in the included studies are summarized in Table 1. There were 2814 cases and 4048 controls. The participants were almost all female with an average age of 37.5 years. The median sample sizes of the cases and controls were 85 and 123, respectively. Most of the studies specified using 1982 or 1997 American College of Rheumatology (ACR) criteria for SLE diagnoses (29 of 33 studies) for cases. The controls included healthy and non-healthy participants with the majority of the studies recruiting healthy controls. Only eight studies recruited samples from the general community. Most studies recruited hospital controls or did not state the source. There were four studies that recruited controls from patients’ relatives.

### VCA (IgG, IgA, and IgM)

**Fig. 1:**
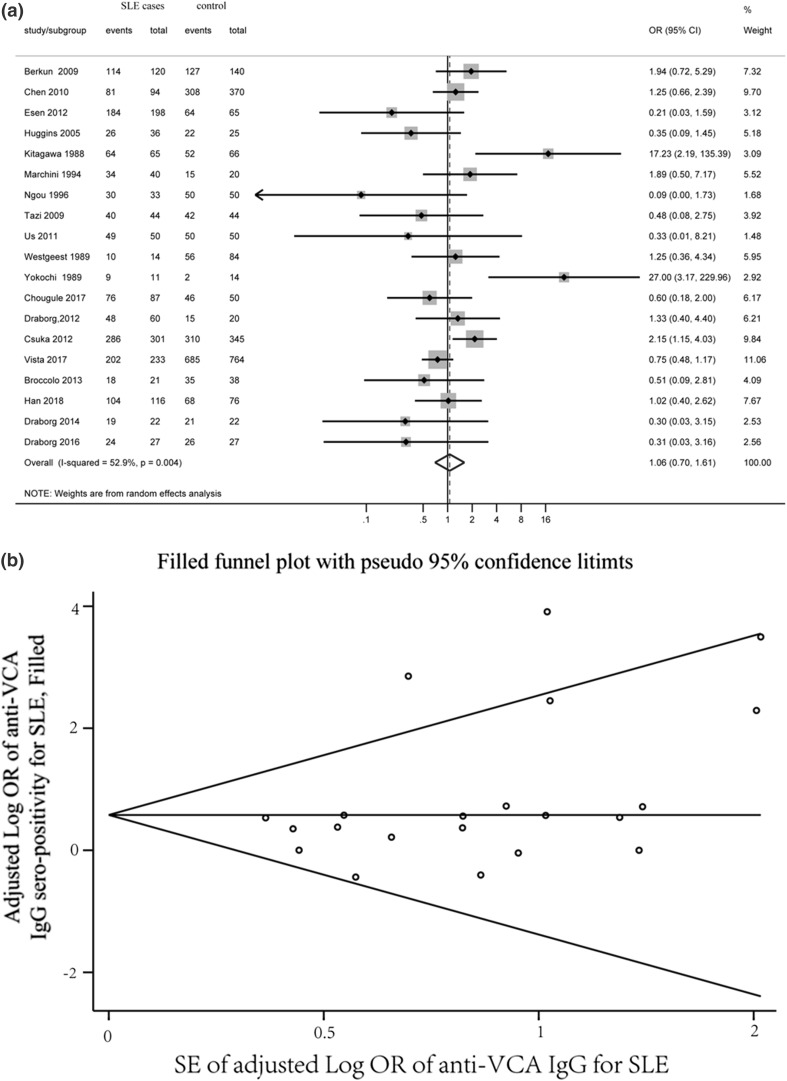
Forest plot of ORs (**a**) and funnel plots with trim and fill (**b**) for anti-VCA IgG sero-positivity. The pseudo 95% confidence interval (CI) is computed as part of the analysis, which produces the funnel plot and corresponds to the expected 95% CI for a given standard error (SE)

There were 20 studies that assayed VCA IgG sero-prevalence. We divided the study of Parks et al. into two separate studies, i.e., African-Americans and whites, making the total number of studies 21. This group found that SLE and the sero-prevalence of EBV antibodies were strongly associated in African-Americans and modestly associated in whites, reflecting significant interaction of race. These studies included a total of 1795 cases and 2635 controls. The mean sero-prevalence of VCA IgG in the cases and controls was 95.0 and 90.8%, respectively. The pooled OR from these studies was 2.06 (95% CI 1.30–3.26, *p *= 0.002) (Fig. 1a). The heterogeneity between studies was significant (*Q* = 40.77, *p* = 0.004, *I*^2^ = 50.9%). Visual inspection of a Begg’s funnel plot revealed asymmetry (*p *= 0.027). We further performed sensitivity analysis using the “trim and fill” procedure to assess the possible effects of publication bias, which involved using unpublished conservative studies to mirror the positive studies that caused plot asymmetry (Fig. 1b). The result demonstrated that no trimming was performed and the data were unchanged, suggesting that the association is not an artifact of unpublished negative studies.

There were six studies that measured VCA IgA, including Parks et al. These studies included a total of 431 cases and 431 controls. The overall percentage of those SLE positive with VCA IgA was higher than that for controls (45.0% and 21.3%, respectively). The pooled OR from these studies was 5.10 (95% CI 2.12–12.28, *p* < 0.001) (Additional file: Figure S2). The heterogeneity between the studies was also significant (*Q* = 18.91, *p* = 0.002, *I*^2^ = 73.6%). Because of the small number of included studies, a funnel plot was not created.

There were eight studies that measured VCA IgM, including Parks et al. These studies included a total of 665 cases and 648 controls. The overall percentage of those SLE positive with VCA IgM was higher than that for controls (21.1% and 8.3%, respectively). The pooled OR from these studies was 2.29 (95% CI 1.34–3.93, *p* = 0.003, *I*^2^ = 20.9%) (Additional file: Figure S3). A funnel plot was not created due to the small number of included studies.

### EBNA (IgG, IgA)

**Fig. 2:**
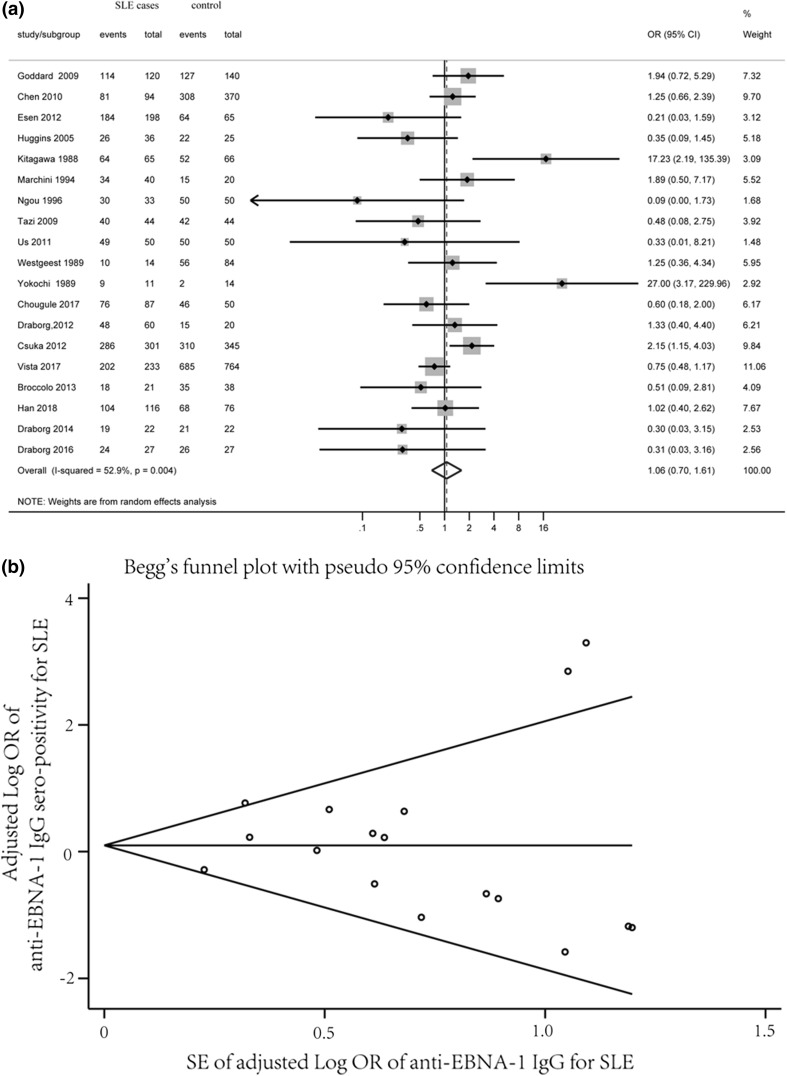
Forest plot of ORs (**a**) and Begg’s Funnel Plot (**b**) for anti-EBNA IgG sero-positivity

We identified 19 studies that tested EBNA IgG sero-positivity, including data for 1572 SLE cases and 2270 controls. The mean sero-prevalence was slightly higher for the SLE group (90.2%) compared with that for controls (87.8%). The estimated pooled OR for EBNA IgG and SLE was 1.06 (95% CI 0.70–1.61, *p *= 0.787), suggesting no significant association between EBNA IgG and SLE (Fig. 2a). The heterogeneity between the studies was moderate (*I*^2^ = 52.9%). Visual inspection of a Begg’s funnel plot revealed symmetry (*p *= 0.484) (Fig. 2b).

Only three studies, including 209 SLE cases and 762 controls, demonstrated EBNA IgA sero-positivity. The estimated pooled OR for EBNA IgA and SLE was 10.40 (95% CI 6.51–16.62, *p *< 0.0001) (Additional file: Figure S4). The heterogeneity between the studies was low (*I*^2^ = 0.0%). A funnel plot was not created due to the small number of included studies.

### EA (IgG, IgA, and IgM)

**Fig. 3:**
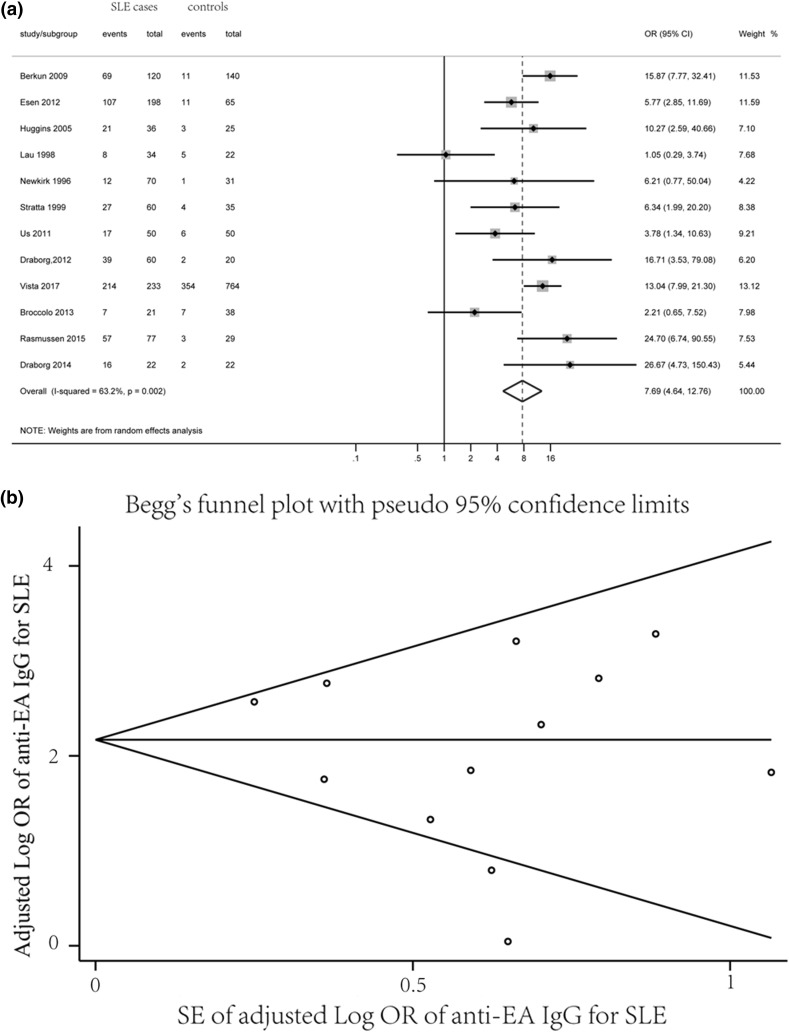
Forest plot of ORs (**a**) and Begg’s funnel plot (**b**) for anti-EA IgG sero-positivity

Twelve studies have reported the sero-positivity of EA IgG between SLE cases and controls. The mean sero-positivity for EA IgG was 60.6% (594/981) for SLE patients and 33.0% (409/1241) for controls. The estimated pooled OR for EA IgG sero-positivity and SLE was 7.70 (95% CI 4.64–12.76, *p *< 0.001) (Fig. 3a). Heterogeneity was moderate (*Q* = 29.9, *p* = 0.002, *I*^2^ = 63.2%). Visual inspection of a Begg’s funnel plot revealed a more symmetrical (*p *= 0.837) distribution of the studies, indicating that publication bias is less likely (Fig. 3b).

There were four studies that assayed the EA IgA sero-prevalence. The prevalence was extremely high in the SLE compared with control groups, 47.2% (91/193) and 4.3% (4/93), respectively. The estimated pooled OR was 16.06 (95% CI 5.99–43.04, *p* < 0.001) with low heterogeneity (*Q* = 1.09, *p* = 0.778, *I*^2^ = 0%) (Additional file: Figure S5).

There were three studies including 159 SLE cases and 71 controls that tested the EA IgM sero-prevalence. The estimated pooled OR was 4.21 (95% CI 2.11–8.40, *p* < 0.001), and heterogeneity was low (*Q* = 1.23, *p* = 0.540, *I*^2^ = 0%) (Additional file: Figure S6).

### Ebv DNA

**Fig. 4:**
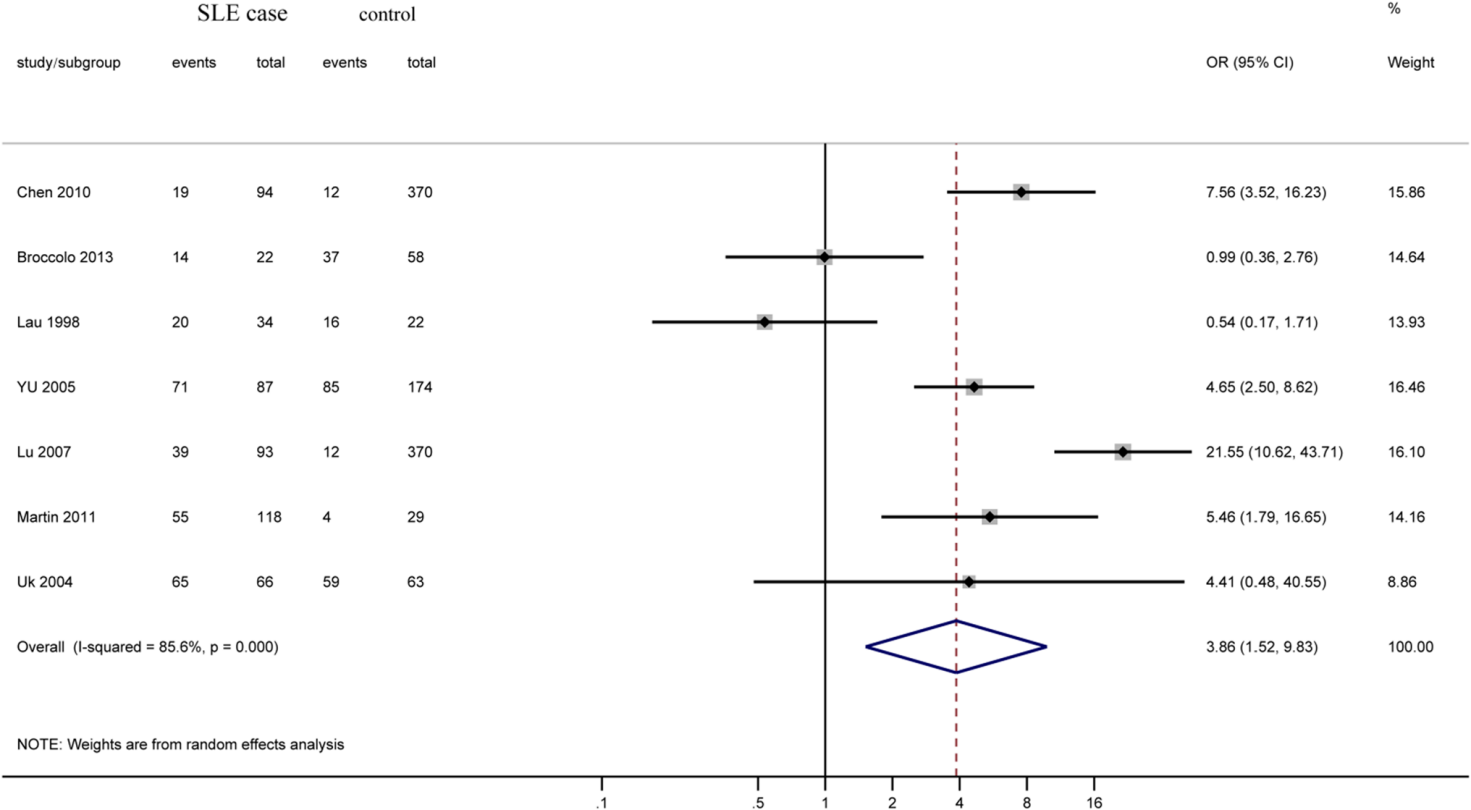
Forest plot of ORs for DNA positivity and SLE

There were seven studies including 514 cases and 1086 controls that tested the EBV DNA-positive rate for the participants. The positive rate for DNA was 55.1% for the SLE group and 20.7% for the control group. Meta-analysis generated an overall OR of 3.864 (95% CI 1.518–9.830, *p *= 0.005) (Fig. 4). Heterogeneity was high with *Q* = 41.74 (*p* < 0.001) and *I*^2^ = 85.6%. Due to the small number of the studies, a funnel plot was not generated.

### Subgroup analysis

**Fig. 5:**
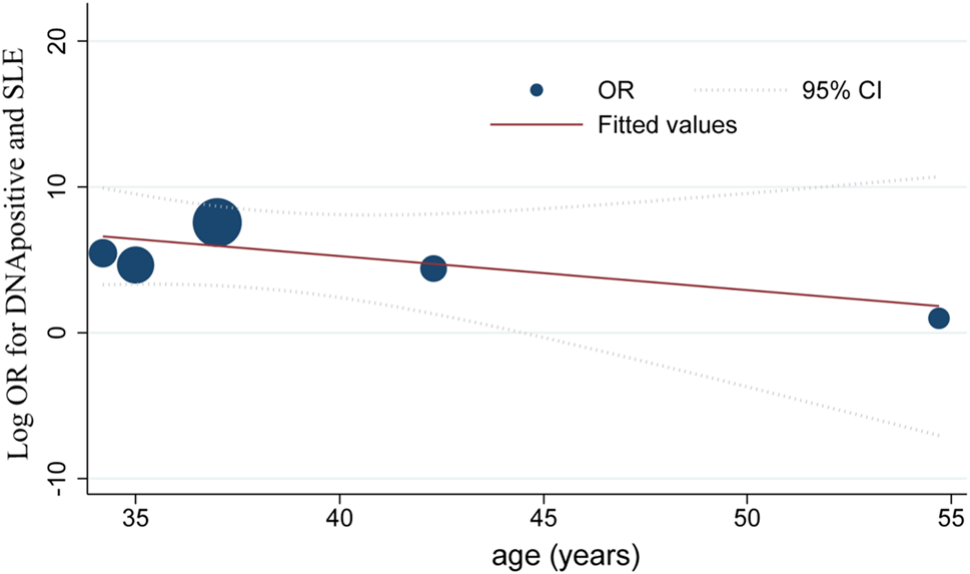
The linear dose–response relationship between the DNA-positive rate and SLE with average age as the explanatory variable. The solid line represents point estimates of the association between EBV DNA positivity and SLE; the dashed lines are 95% CIs. Circles present the dose-specific OR estimates reported in each study. The area in each circle is proportional to the inverse variance of the OR. The vertical axis is on a log scale

To explore study heterogeneity, we performed subgroup analysis across a number of significant study characteristics. Age, sex, living place, diagnosis criteria for the cases, source of controls, and method for detecting antibodies were not a significant source of heterogeneity (Table 2). There was a trend that pediatric patients had higher ORs compared with adult patients, but there was no significant difference. According to some major studies, age was a significant confounding factor. We undertook an exploratory meta-regression analysis of the average age in each study. The results demonstrated that the average age significantly correlated with DNA positivity and SLE (*p *= 0.004) (Fig. 5).

**Table 2:** Summary of subgroup analysis results

Specified for antibody or DNA	Matching (95% CI)	Not matching (95% CI)	Difference significance (*p* value)
Matching and not matching for age			
VCA IgG	2.82 (1.38–5.75)/12 studies	2.06 (1.31–3.26)/9 studies	0.92
EBNA IgG	0.86 (0.52–1.44)/7 studies	1.27 (0.71–2.27)/12 studies	0.24
EA IgG	5.50 (1.24–24.28)/4 studies	8.96 (5.83–13.78)/8 studies	0.42
DNA	7.14 (3.47–14.70)/5 studies	3.25 (0.94–11.30)/2 studies	0.60
Matching for sex			
VCA IgG	1.59 (0.98–2.59)/6 studies	2.39 (1.21–4.72)/15 studies	15
EBNA IgG	0.81 (0.58–1.13)/6 studies	1.32 (0.72–2.40)/13 studies	13
EA IgG	5.50 (1.24–24.28)/4 studies	8.96 (5.83–13.78)/8 studies	8
DNA	3.53 (1.09–11.43)/5 studies	5.23 (1.93–4.16)/2 studies	2
Matching for living place			
VCA IgG	1.52 (0.98–2.34)/7 studies	2.46 (1.20–5.05/14 studies	0.51
EBNA IgG	1.23 (0.74–2.05)/5 studies	0.94 (0.49–1.81/12 studies	0.54
EA IgG	9.08 (3.78–21.84/3 studies	7.12 (3.82–13.30)/9 studies	0.74
DNA	5.65 (1.10–28.98/3 studies	3.86 (1.52–9.83)/4 studies	0.47

Specified for antibody or DNA	IFA (95% CI)	ELISA (95% CI)	Difference significance (*p* value)
Test method for serum antibodies			
VCA IgG	1.61 (0.82–3.17)/9 studies	2.64 (1.36–5.11)/12 studies	0.32
EBNA IgG	1.41 (0.40–4.95)/7 studies	1.02 (0.74–1.41)/12 studies	0.38
EA IgG	6.95 (3.29–14.72)/6 studies	8.56 (3.91–18.71)/6 studies	0.71

Specified for antibody or DNA	Pediatric studies (95% CI)	Adult studies (95% CI)	Difference significance (*p* value)
Pediatric vs. adult studies			
VCA IgG	3.88 (0.08–187.98)/2 studies	2.04 (1.29–3.22)/19 studies	0.93

Specified for antibody or DNA	1997 ACR criteria for SLE (95% CI)	1982 ACR criteria or unspecified (95% CI)	Difference significance (*p* value)
Diagnosis criteria for cases			
VCA IgG	2.01 (0.95–4.27)/12 studies	1.92 (1.15–3.22)/9 studies	0.82
EBNA IgG	0.82 (0.55–1.22)/10 studies	1.72 (0.85–3.49)/9 studies	0.03
EA IgG	9.61 (5.05–18.28)/6 studies	5.75 (2.51–13.19)/6 studies	0.33

Specified for antibody or DNA	High score (95% CI)	Low score (95% CI)	Difference significance (*p* value)
Quality of studies			
VCA IgG	2.11 (1.23–3.61)/16 studies	2.03 (0.76–5.45)/5 studies	0.99
EBNA IgG	0.89 (0.67–1.20)/10 studies	1.49 (0.62–3.54)/9 studies	0.16
EA IgG	9.33 (5.53–15.74)/7 studies	5.60 (1.88–16.73)/5 studies	0.36
DNA	5.45 (1.81–16.48)/3 studies	2.19 (0.40–11.84)/3 studies	0.37

### Quality assessment

According to the modified NOS scale, the maximum score that could be achieved by a study was 12 stars. In our meta-analysis, the median score for all studies was five. The highest was rewarded by Parks et al. with nine stars. For selection criteria, only two studies did not specify a definition for the cases. However, only 6 of 33 recruited cases for consecutive or representative patients. Eight studies selected adequate controls from the community. For comparability criteria, 15 studies did not match cases and controls with confounders. Ten out of the remaining 18 studies matched for age and at least one additional factor. As for exposure, few studies reported the blinding of analyses or missing data. About half of the studies listed cutoffs for the assays.

To examine the influence of the quality of studies on ORs, we compared studies with higher NOS scores (equal to or above the median of the overall studies) to studies scoring below the median in a post hoc analysis. The ORs were higher for all EBV IgG and DNA outcomes in the higher scoring studies with the exception of EBNA IgG. However, there was no statically significant difference (Table 2).

## Discussion

Our review has again found an association between EBV sero-positivity and SLE based on VCA antibody (IgG, IgA, IgM), EBNA IgA, and EA antibody (IgG, IgA, IgM) testing. We did not observe evidence of differences in the sero-prevalence of EBNA IgG, which is indicative of latent infection. This analysis shows a significant association between the EBV DNA-positive rate and SLE (OR: 3.86, 95% CI 1.52–9.83, *p *= 0.005). Furthermore, meta-regression demonstrates that the average age of the participants negatively correlated with the association between DNA positivity and SLE (*p *= 0.004). To our knowledge, this systemic review is the first attempt to combine such estimates of the association between SLE and EBV DNA positivity.

Hanlon et al. [6] included 25 studies in their meta-analysis, but four of the studies did not specify the antigen for the tested antibody. Therefore, only 21 of these studies were used for analysis. In our review, 12 additional studies were added for analysis. In addition to increasing the total number of cases, the average sample size also increased. As a matter of fact, our results even more precisely verify Hanlon’s findings.

There was considerable heterogeneity between studies, and we examined different factors that might have been influential. Although none of the subgroup analyses reached statistical significance. Slightly higher OR values were observed for the pediatric studies with a wide confidence interval (0.08–187.98) compared with adult studies. James et al. [8] found that EBV infection increases SLE risk by as much as 50-fold in children, while Tsai et al. [10] found no difference between SLE patients and healthy controls. This discrepancy may be due to the small number of pediatric studies (only two) or only chance.

We could not find any difference in the combined OR of the studies using more recent criteria (1997 ACR criteria for SLE) compared with older criteria (1982 ACR criteria for SLE) or failure to report criteria (which may reflect a lack of reporting). Studies matched for age, sex, or living place did not have different ORs for EBV IgG sero-positivity. Other potential vital confounders include ethnicity and socioeconomic status. We did not test the effects of controlling for these factors because few studies have specifically mentioned controlling for them. Parks et al. [29] reported a racial difference in the association between EBV IgA and SLE where African-Americans were more likely to have a history of EBV infection. Comparing the ORs of studies that used IFA to detect EBV antibodies vs. those that used ELISA revealed a similar result. However, Almohmeed et al. [45] demonstrated that using IFA to test VCA and EA IgG had twice the OR compared with ELISA for MS. Because there is a lack of detailed studies analyzing the two testing methods for SLE, we could not explain the discrepancy between these two autoimmune diseases. Other elements that may contribute to differences in estimates of ORs include the source and selection of patients and controls. Few studies have clearly described their methods for selecting cases (random or consecutive). Ideally, society would be the best source for controls. However, only eight studies have been deemed to have appropriate community controls [16, 18, 29, 33, 40–42, 44]. There were six studies that recruited bone marrow or blood donors [15, 17, 22, 28, 30, 31], which may be considered inappropriate community controls because such donors were selected with unhealthy risk behavior. There were four studies that recruited relatives of patients [8, 23, 35, 36], which are also inappropriate controls as relatives may share the same susceptible genetic factors as patients. Sixteen studies have recruited only seemingly healthy controls, and we observed that nine of these studies found no statistically significant higher EBV IgG sero-prevalence in cases compared with controls [19, 26–29, 34, 37, 41, 42].

The pooled OR for EBV DNA and SLE was 3.86, which is consistent with sero-antibody prevalence. Yu et al. [39] reported a high OR for an SLE patient that was significantly different from others because the controls had low positivity in serum samples. Although three of seven studies failed to find a significant difference in the DNA-positivity rate and SLE [9, 37, 44], and one found a high DNA load in SLE compared with controls [44]. The negative result may be due to chance or the small sizes of these studies. Yu et al. [39] reported that the EBV DNA-positive rate declines with age for controls but not for SLE patients. Although our subgroup analysis did not find a significant difference between age-matched and unmatched groups, meta-regression demonstrated that the OR for the DNA-positive rate declines with average age. This may be due to the fact that young SLE patients tend to be more infected by EBV, which is consistent with James et al. [8].

EBV subclinically infects a majority of individuals worldwide and generates multiple antibodies in the serum. The most common antibody is VCA IgG, which represents exposure to EBV and lasts lifelong. Our studies found that the overall prevalence of VCA IgG in SLE patients and controls was 95.0 and 90.8%, respectively, and the OR reached a significant difference. In addition, many studies have revealed a higher titer in SLE patients compared with controls [18, 23, 33–35]. Otherwise, the best way to specify the relationship between SLE and EBV infection is at the DNA level, which also had a higher OR in our study. A conclusion may be drawn that prior EBV infection is an essential factor for the development of SLE. Many studies had found that some humoral autoimmunity in SLE arises from molecular mimicry between EBNA-1 and lupus auto-antigens, which provide further evidence for suspecting an etiologic role for EBV in SLE. However, our study failed to find a higher prevalence of EBNA IgG in SLE patients, which is difficult to explain. Furthermore, a prospective study with a large sample size should be considered to solve this enigma. Antibodies directed against EA/D are generally known as an indication of lytic replication. The sero-prevalence of these antibodies is significantly higher for SLE patients, particularly for IgA, compared with controls, and the ORs also achieved statistical significance. The majority of the included studies have been consistent on this issue. Draborg et al. [33] thought that a possible mechanism for this phenomenon as a specific intrinsic defect in the immune systems of SLE patients is independent of immunosuppressive medication therapy.

The strengths of this meta-analysis include its comprehensive search strategy and absence of language restrictions; however, limitations must also be considered. First, the quality of individual studies was not always ideal, as demonstrated by a general lack of information on the recruitment of consecutive patients and selection controls for all studies. Second, there is heterogeneity in the ORs across studies, and subgroup analysis did not reveal the source. This heterogeneity may be derived from other confounders, such as ethnicity and laboratory measurements. Third, some of the funnel plot analyses showed asymmetry that suggested the possibility of publication bias. The trim and fill sensitivity analysis did not modify the general results, suggesting that association is not an artifact of unpublished negative studies. Nevertheless, that possibility is not entirely excluded by this method. Fourth, similar to all meta-analyses, our study has the limitation of being a retrospective analysis; thus, further prospective cohort studies are warranted to confirm these findings.

In conclusion, our review supports the hypothesis that prior EBV infection is an essential factor for the development of SLE as indicated by a higher positivity of VCA IgG and EBV DNA. These findings also suggest abnormal (EA, IgA, EBV DNA) humoral immune responses to EBV in the context of SLE. However, the studies included in our meta-analysis are heterogeneous and have small sample sizes. At the same time, many studies did not match for age and gender, only a few matched for race and other confounding factors, and descriptions of recruitment and laboratory testing were not specified in most of the papers. Moreover, the role of publication bias could not be excluded. Large prospective studies are needed to determine the relationship between SLE and infection before we could draw a causal relationship between the two.

## Electronic supplementary material

Below is the link to the electronic supplementary material.

Supplementary material 1 (DOC 1114 kb)

## List of abbreviations

EBV: Epstein–Barr virus
SLE: Systemic lupus erythematosus
VCA: Viral capsid antigen
EA: Early antigen
EBNA: Epstein–Barr nuclear antigen
OR: Odds ratio
NOS: Newcastle–Ottawa scale
ACR: American College of Rheumatology
PBMCs: Blood mononuclear cells
ELISA: Enzyme-linked immuno sorbent assay
PCR: Polymerase chain reaction
IFA: Indirect immunofluorescence assay
