# Supplementary material for: The risk of systemic lupus erythematosus associated with Epstein–Barr virus infection: a systematic review and meta-analysis
Source: Clin Exp Med. 2018 Oct 25;19(1):23–36. doi: 10.1007/s10238-018-0535-0 (PMC6394567; doi:10.1007/s10238-018-0535-0)
Supplement: Supplementary file 1 — Supplementary material 1 (DOC 1114 kb) [file 10238_2018_535_MOESM1_ESM.doc]

1221 titles and abstracts screened once duplicates removed

63 studies identified and full texts obtained

30 studies excluded

18 had no or inadequate EBV serology

10 lacked control group

1 diagnosis other than SLE

1 data already reported in other studies

12 had no control group

33 studies eligible for inclusion

**Initial search:**

Medline: 755 records

EMBASE:1245 records

**Additional papers:**

One additional study was identified from citation searching/reference lists

**Figure S1. Flow diagram of literature search and study selection.**

**Table S1 - MeSH/ Emtree headings and text words in search strategy (SLE terms combined with EBV terms)**

| **Database** | **Systemic lupus erythematosus** | **Epstein Barr virus** |
| --- | --- | --- |
| **MEDLINE MeSH Headings** | 1. exp Lupus Erythematosus, Systemic 2. exp Lupus Nephritis 3. exp Lupus Vasculitis, Central Nervous System | 1. exp Epstein-Barr Virus Infections  2. exp Herpesvirus 4, Human  3. exp Infectious Mononucleosis |
| **Embase Emtree headings** | 1. exp Lupus Erythematosus, Systemic 2. exp Lupus Nephritis 3. exp Lupus Vasculitis, Central Nervous System | 1. exp Epstein-Barr Virus Infections  2. exp Herpesvirus 4, Human  3. exp Infectious Mononucleosis |
| **Text words (used in both databases)** | 1. Systemic Lupus Erythematosus.tw. 2. Lupus Erythematosus Disseminatus.tw. 3. Libman-Sacks Disease.tw. 4. Disease, Libman-Sacks.tw. 5. Libman Sacks Disease.Central Nervous System Lupus Vasculitis.tw. 6. Systemic Lupus Erythematosis, Central Nervous System.tw. 7. Central Nervous System Lupus.tw. 8. Central Nervous System.tw. 9. Systemic Lupus Erythematosis.tw. 10. Neuropsychiatric Systemic Lupus Erythematosus.tw. 11. Lupus Meningoencephalitis.tw. 12. Lupus Meningoencephalitides.tw. 13. Meningoencephalitides, Lupus.tw. 14. Meningoencephalitis, Lupus.tw. 15. Lupus Glomerulonephritis.tw. 16. Nephritis, Lupus.tw. 17. Lupus Nephritides.tw. 18. Nephritides, Lupus.tw. 19. Glomerulonephritis, Lupus.tw. 20. Glomerulonephritides, Lupus.tw. 21. Lupus Glomerulonephritides.tw. | 1. epstein barr.tw 2. EBV.tw 3. Mononucleosis.tw 4. glandular fever.tw 5. herpesvirus 4.tw or HHV 4.tw 6. kissing disease.tw |

**Table S2: The quality assessment of included case-control studies using the Newcastle-Ottawa scale.**

|  | **Selection** | | | | **Comparability** | | **Exposure** | | | | |  |
| --- | --- | --- | --- | --- | --- | --- | --- | --- | --- | --- | --- | --- |
| Author | S1 | S2 | S3 | S4 | C1 | C2 | E1a | E1b | E2 | E3 | E4 | Total |
| Berkun 2009 | * | - | * | * | * | * | - | - | - | * | - | 6 |
| Chen 2005 | * | - | - | - | * | * | - | - | * | * | - | 5 |
| Chen 2010 | * | * | - | * | - | - | - | - | * | * | - | 5 |
| Esen 2012 | * | * | - | * | - | - | - | - | * | * | - | 5 |
| Huggins 2005 | * | - | - | * | - | - | - | - | - | * | - | 3 |
| James 1997 | * | - | - | - | * | * | - | - | * | * | - | 5 |
| James 2001 | * | - | - | * | * | * | - | - | * | * | - | 6 |
| Kitagawa 1988 | * | - | - | - | - | - | - | - | - | * | - | 2 |
| Lau 1998 | * | - | - | - | - | - | - | - | - | * | - | 2 |
| Lu 2007 | * | - | - | - | * | * | - | - | * | * | - | 5 |
| Marchini 1994 | - | - | - | - | - | - | - | - | - | * | - | 1 |
| Newkirk 1996 | * | - | - | * | - | - | - | - | - | * | - | 3 |
| Ngou 1996 | * | - | - | - | - | - | - | - | - | * | - | 2 |
| Parks 2005 | * | * | * | - | * | * | ** | n/a | * | * | - | 9 |
| Stratta 1999 | * | - | - | * | - | * | - | * | - | * | - | 5 |
| Tazi 2009 | * | * | - | * | * | * | - | - | * | * | * | 8 |
| Tsai 1995 | * | - | - | * | * | - | - | - | * | * | - | 5 |
| Us 2011 | * | - | - | * | - | - | - | - | - | * | - | 3 |
| Westgeest 1989 | * | - | - | * | - | - | - | - | - | * | - | 3 |
| Yokochi 1989 | * | - | - | * | - | - | - | - | - | * | - | 3 |
| Zhang 1999 | - | - | * | * | - | - | - | - | * | * | * | 5 |
| Draborg 2012 | * | - | * | * | - | - | - | - | * | * | - | 5 |
| Chougule 2017 | * | - | - | - | - | - | - | - | - | * | - | 2 |
| Csuka 2012 | * | - | - | - | - | - | - | - | * | * | - | 3 |
| Vista 2017 | * | - | - | * | * | * | - | - | * | * | - | 6 |
| Broccolo 2013 | * | - | - | - | * | * | - | - | * | * | - | 5 |
| Han 2018 | * | * | - | * | * | - | - | - | - | * | - | 5 |
| YU 2005 | * | - | - | * | * | * | - | - | * | * | - | 6 |
| Rasmussen 2015 | * | - | * | - | - | - | - | - | - | * | - | 3 |
| Draborg 2014 | * | - | * | - | * | * | - | - | * | * | - | 6 |
| Draborg 2016 | * | - | * | - | * | * | - | - | - | * | - | 5 |
| Martin 2011 | * | * | - | - | - | - | - | - | - | * | - | 3 |
| Uk 2004 | * | - | * | - | - | - | - | - | - | * | - | 3 |

The quality of the included studies was assessed by the Newcastle Ottawa scale. A study can be awarded a maximum of one star for each numbered item within the Selection and Exposure categories and a maximum of two stars for Comparability.

**Abbreviations:** S1: Adequate definition of cases, S2: Consecutive or obviously representative cases, S3: Adequate community controls, S4: controls specified as having no history of disease**,** C1: Matching for age, C2: Matching for additional factors, E1 a: Blinding of sample analysis, E1 b: Conducted analysis in clinical laboratory, E2: Explicit serology cut-off values reported, E3: Same serology method used for cases and controls, E4: Missing data reported


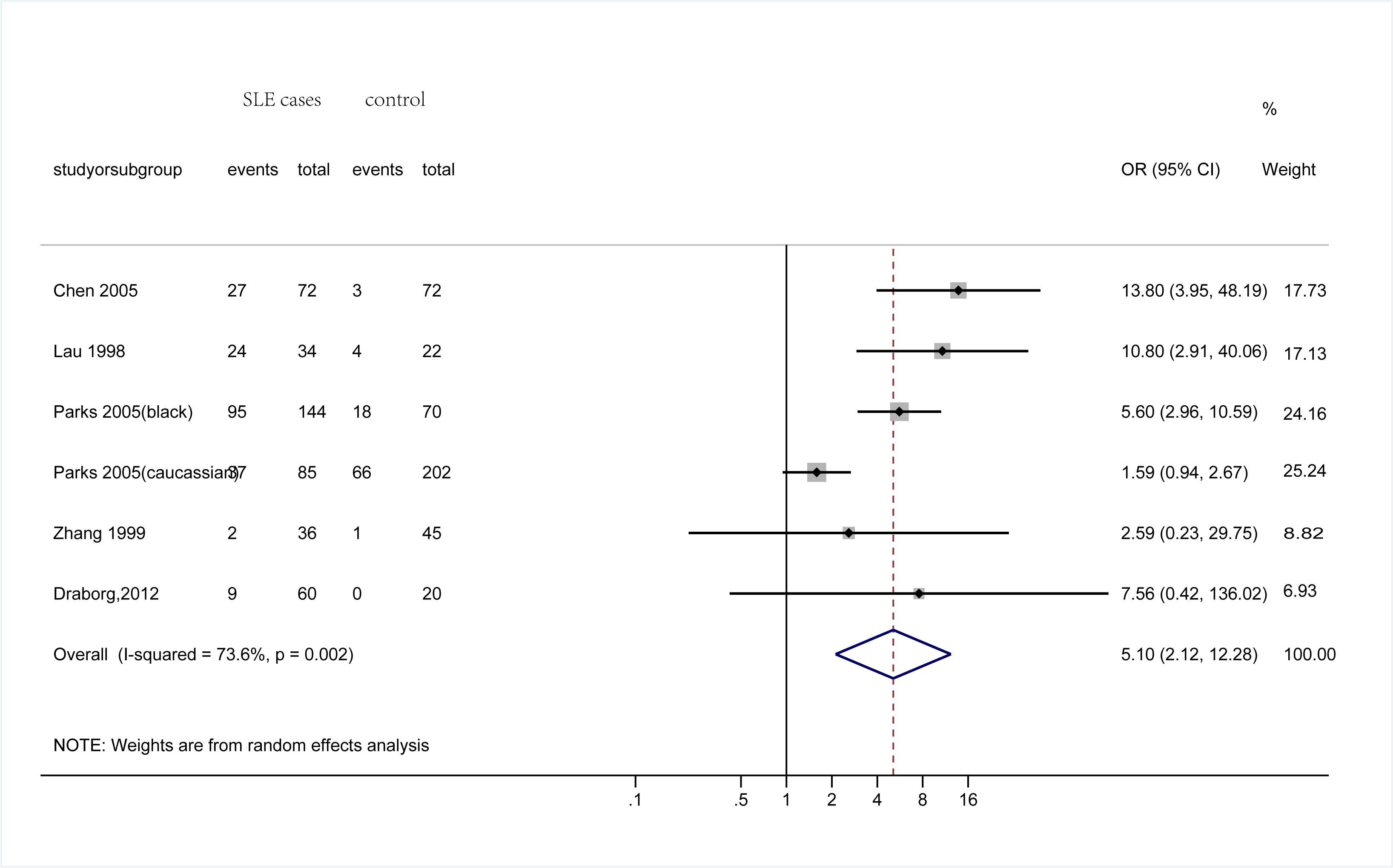


Figure S2. Forest plots of ORs for anti-VCA IgA and SLE


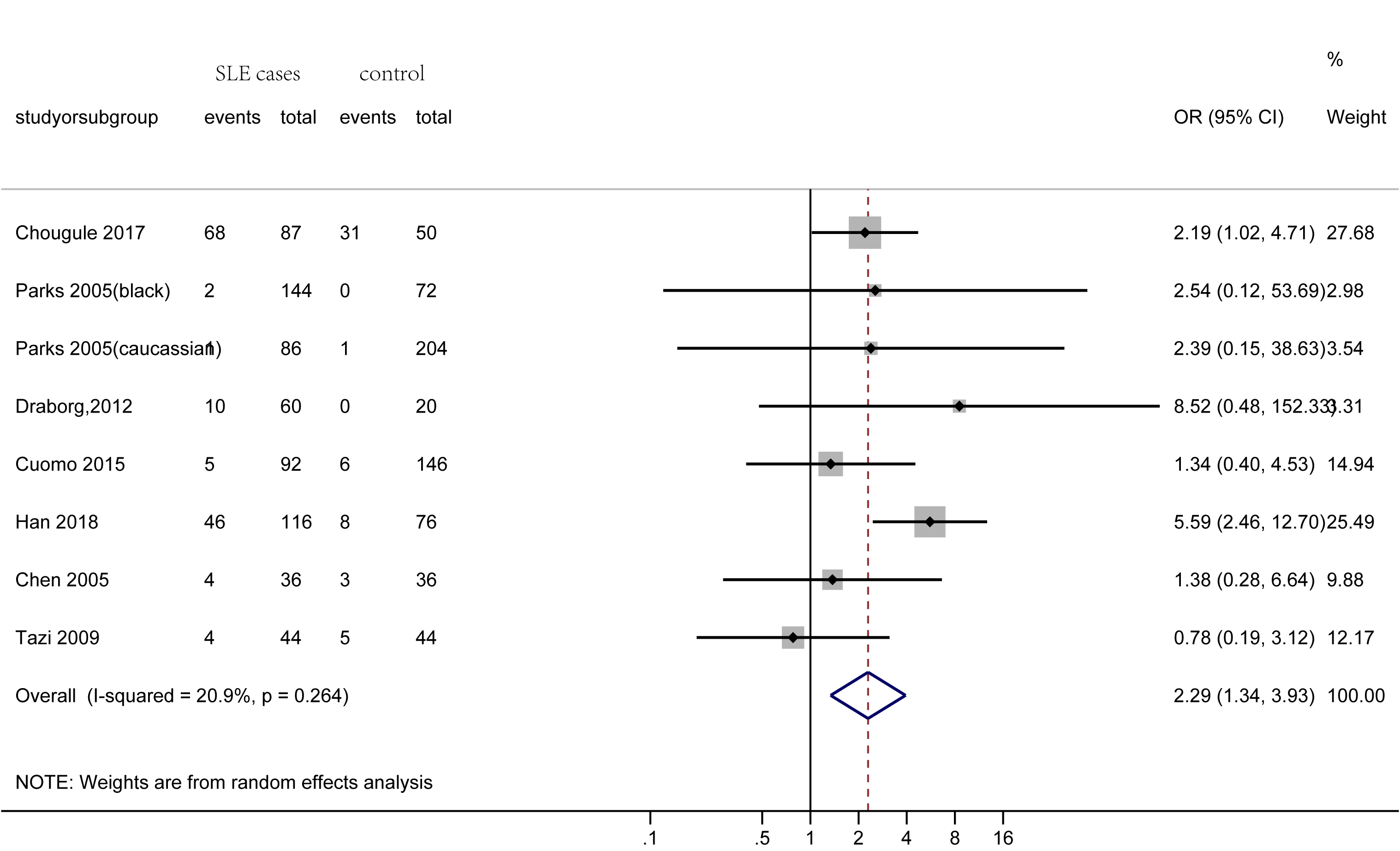


Figure S3. Forest plots of ORs for anti-VCA IgM and SLE


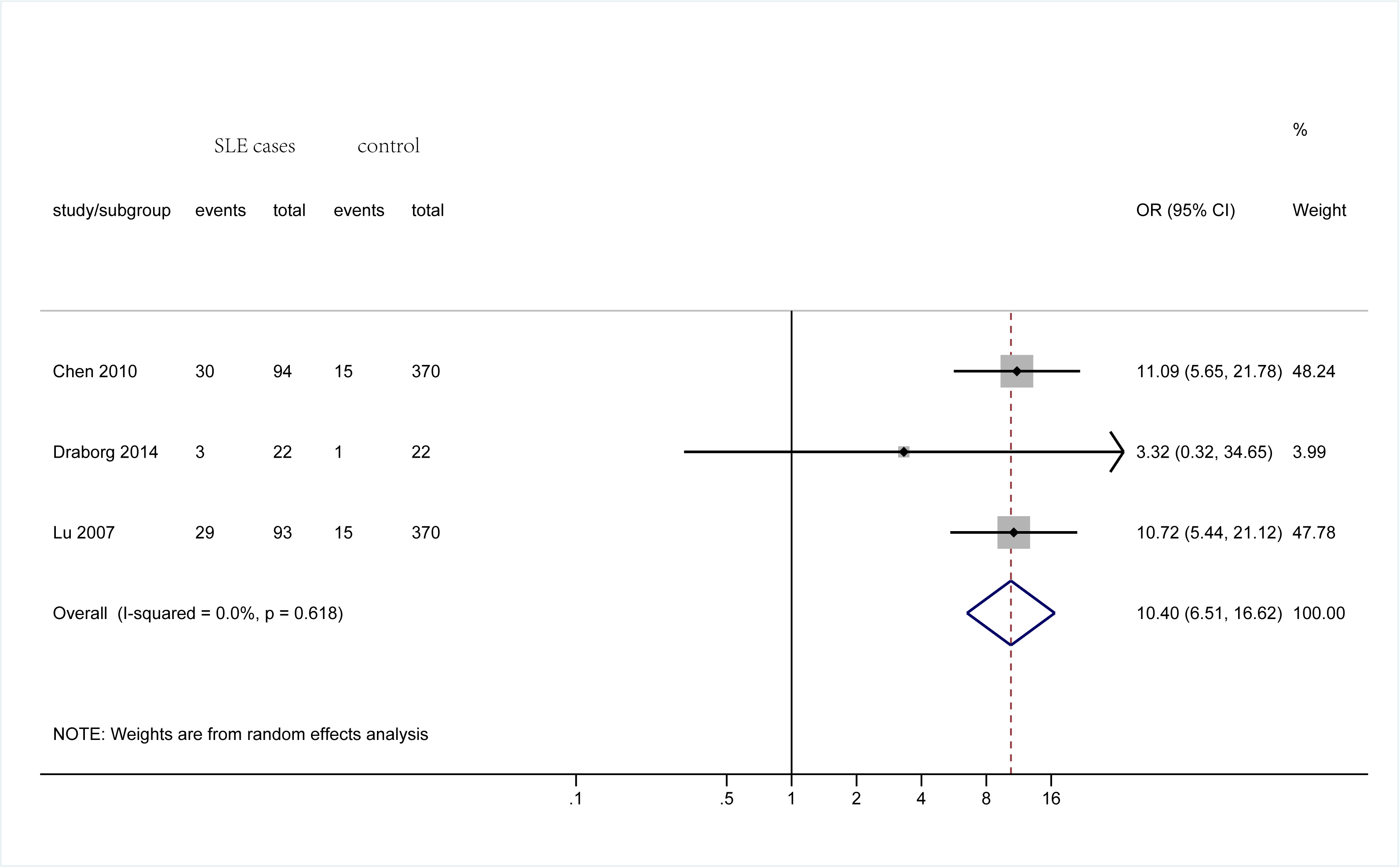


Figure S4. Forest plot of ORs for anti-EBNA IgA and SLE


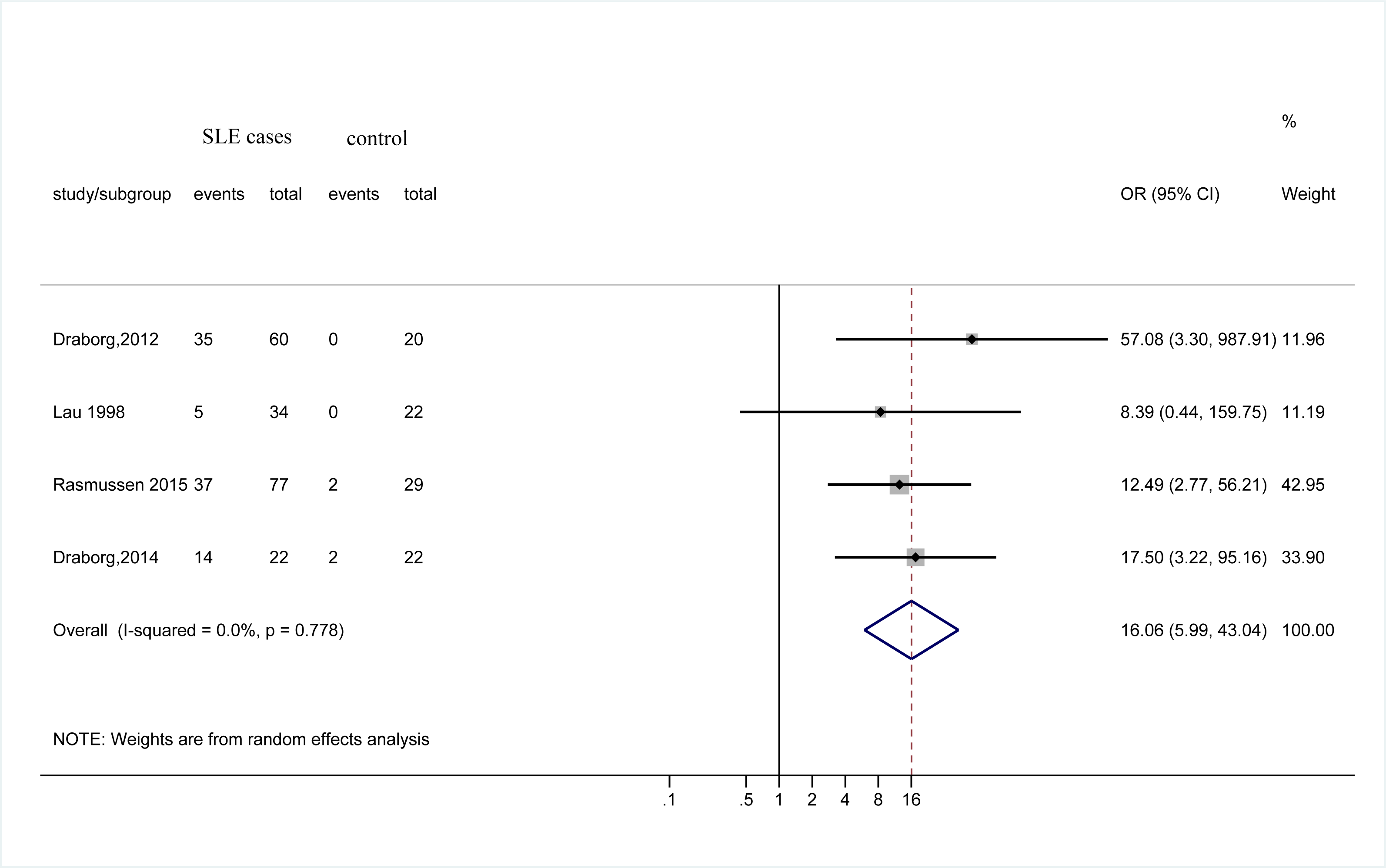


Figure S5. Forest plots of ORs for anti-EA IgA and SLE


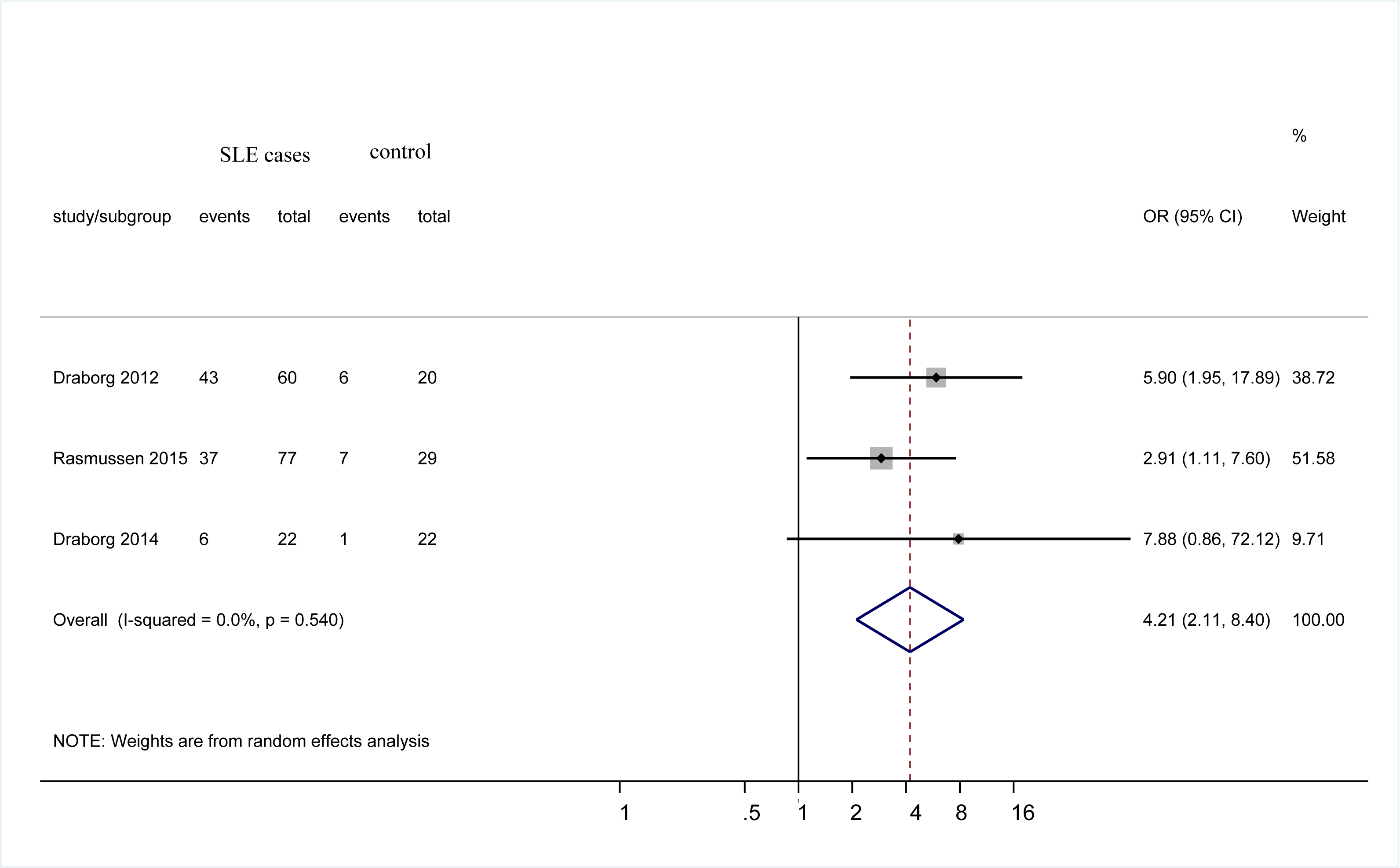


Figure S6. Forest plots of ORs for anti-EA IgM and SLE
